# The influence of the gut microbiome on ovarian aging

**DOI:** 10.1080/19490976.2023.2295394

**Published:** 2024-01-03

**Authors:** Feiling Huang, Ying Cao, Jinghui Liang, Ruiyi Tang, Si Wu, Peng Zhang, Rong Chen

**Affiliations:** a Department of Obstetrics and Gynecology, Peking Union Medical College Hospital, Chinese Academy of Medical Sciences & Peking Union Medical College, National Clinical Research Center for Obstetric & Gynecologic Diseases, Beijing, China; b School of Medicine, Hunan Normal University, Changsha, Hunan, China; c Beijing Key Laboratory for Genetics of Birth Defects, Beijing Pediatric Research Institute; MOE Key Laboratory of Major Diseases in Children; Rare Disease Center, Beijing Children’s Hospital, Capital Medical University, Beijing, China

**Keywords:** Ovarian aging, menopause, premature ovarian insufficiency, gut microbiome, fecal microbiota transplantation, anti-ovarian aging

## Abstract

Ovarian aging occurs prior to the aging of other organ systems and acts as the pacemaker of the aging process of multiple organs. As life expectancy has increased, preventing ovarian aging has become an essential goal for promoting extended reproductive function and improving bone and genitourinary conditions related to ovarian aging in women. An improved understanding of ovarian aging may ultimately provide tools for the prediction and mitigation of this process. Recent studies have suggested a connection between ovarian aging and the gut microbiota, and alterations in the composition and functional profile of the gut microbiota have profound consequences on ovarian function. The interaction between the gut microbiota and the ovaries is bidirectional. In this review, we examine current knowledge on ovary-gut microbiota crosstalk and further discuss the potential role of gut microbiota in anti-aging interventions. Microbiota-based manipulation is an appealing approach that may offer new therapeutic strategies to delay or reverse ovarian aging.

## Introduction

The gut microbiota, which is the second genome of the human body, plays an important role in human health and pathogenesis. The gut microbiota is a complex symbiotic ecosystem, and imbalances in the gut microbiota have been widely reported in a range of common chronic diseases, such as type 2 diabetes, cardiometabolic diseases and metabolic liver diseases.^1^ Mounting evidence has confirmed that there is a bidirectional relationship between the gut and numerous crucial organs of the host, in which the microbiota serves as a regulator.^2,3^

Significant changes in the identities of dominant microorganisms, bacterial diversity, and functional features have been uncovered in human aging and age-related diseases.^4,5^ A recent study showed the existence of multiple “clocks” within the body: organs/systems age at different rates, and the aging rate of specific organs or systems correlates with that of the gut microbiome.^6^ Consequently, individuals may present with different health or disease states. Gut microbial features can be used to predict healthy aging or mortality in elderly individuals.^7,8^ Interestingly, experiments on mice demonstrated that transplantation of aged donor microbiota accelerates specific processes of age-associated brain degeneration in the recipient, and conversely, transplantation of young donor microbiota can reverse this condition.^9,10^ It has been suggested that the gut microbiota could serve as a potential therapeutic target for new anti-aging interventions.^5^

**Figure 1.**
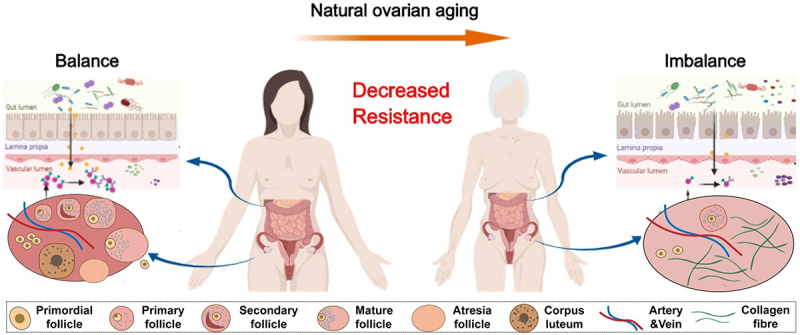
The physiologic succession of gut microbiota across natural ovarian aging.

Ovarian aging refers to the gradual decline in ovarian function with age. It is characterized by the gradual deterioration in oocyte quantity and quality, accompanied by menstrual cycle irregularity, infertility and finally, the cessation of menses.^11^ The ovary experiences a different lifespan from other organs. Recently, multiomics profiling identified a significant acceleration in female biological aging around the third and fifth decades of life, which coincided with the time points of fertility decline and menopausal status.^12^ Thus, ovarian aging is considered the pacemaker of aging in the female body, driving the aging of multiple organs.^13^ The levels of sex hormones, including follicle-stimulating hormone (FSH), luteinizing hormone (LH) and anti-Müllerian hormone (AMH), were highly correlated with several other aging clocks.^12^ As life expectancy increases worldwide, ovarian aging will gradually become a major health problem for women.^14^ Ovarian aging is an extremely complex process with causes that have not yet been fully clarified. Growing evidence shows that there is a link between the gut microbiota and ovarian function,^15^ and the gut microbiota-ovary axis contributes to follicular development.^16^ In this review, we aim to synthesize different aspects of ovary-microbiota crosstalk and provide meaningful conclusions (Figure 1). We also try to explain the potential role of the microbiota in some anti-aging approaches.

## The gut microbiota and natural ovarian aging

Menopause is the hallmark event of natural ovarian aging. Natural menopause, which mostly occurs between the ages of 49 and 52 years, is diagnosed retrospectively after a 12-month cessation of spontaneous menses without pathological causes.^17^ As the finite stores of ovarian follicles are depleted and the remaining follicles lose their sensitivity to gonadotropins, the follicles stop developing and secreting the ovarian hormones estrogen and progesterone.^18^

Estradiol (E2) levels only decrease relatively late during the process of perimenopausal transition.^11^ However, a dramatic decline in plasma E2 levels occurs during the final menstrual cycle, and afterward, the postmenopausal ovaries stop synthesizing E2.^19^ It is known that the gut microbiome is one of the important regulators of circulating estrogens.^20^ Gut microbial β-glucuronidase (gmGUS) can transform estrogen from deactivated forms to active forms and further affect estrogen levels in the host.^21,22^ Gut microbiota dysbiosis and lower microbial diversity result in a reduction in gmGUS and alterations in systemic estrogens.^15^ Gut bacteria, in turn, are also influenced by estrogen. It has been reported that the relative abundances of two bacterial taxa, namely, the *Gammaproteobacteria* class and an unknown family from *Mixococcales*, were positively correlated with E2 levels, and the abundance of the family *Prevotellaceae* was negatively correlated with E2 levels.^23,24^ Furthermore, estrogen or estrogen-like compounds exert microbiome-modulating effects and maintain gut epithelial barrier integrity in mice with metabolic syndrome.^25^ Supplementation with estrogen-like foods such as soy isoflavones augments the abundances of the genera *Bifidobacterium* and *Eubacterium* while restraining the genera *Lactobacillus* and unclassified *Clostridiaceae* in postmenopausal women.^26^ Under certain pathological conditions, dysbiosis of the gut microbiota and elevated gmGUS activity may contribute to the development of estrogen-driven diseases and menopause-related disorders by participating in the circulation or activation of endogenous and exogenous estrogen-like compounds.^21^

Menopausal status is known to affect the gut microbiota. Differences in the composition and the functional profile of the gut microbiota between women of different menopausal statuses have already been reported as described below. A total of 90 differentially abundant taxa between premenopausal (*n* = 44) and postmenopausal women (*n* = 45) were observed, and the pyrimidine and one-carbon pools associated with the folate pathways were enriched in premenopausal women compared with postmenopausal women.^27^ The genus *Roseburia* was identified as the most remarkable bacterial taxon to discriminate between premenopausal (*n* = 17) and postmenopausal groups (*n* = 20), and higher propanoate and butanoate metabolism in premenopausal women was observed.^23^ In contrast, Zhao et al. demonstrated that the alpha diversity of microbiomes in postmenopausal women (*n* = 24) was significantly lower than that in premenopausal women (*n* = 24), the abundances of the phylum *Firmicutes* and the genus *Roseburia* were low, the abundances of the phylum *Bacteroidetes* and the toluene-producing genus *Tolumonas* were enriched in postmenopausal women, and homocysteine synthesis-related processes were activated in postmenopausal women.^28^ Other microbes, such as the genera *A. odoratum* and *B. cholerae*, were also enriched in postmenopausal women (*n* = 90) compared with premenopausal women (*n* = 66).^24^ Remarkably, *Firmicutes* and *Bacteroidetes* are dominant groups of beneficial bacteria in the human gut^29^ and *Roseburia* is a butyrate-producing bacteria.^30,31^ Although taxa across studies varied, it might be attributed to the different study populations, sample sizes or methods.

In an animal model, alterations in the gut microbiota induced by ovarian aging were also described. 4-Vinylcyclohexene diepoxide (VCD), an occupational chemical that directly kills primordial and primary ovarian follicles of the ovary but shows no toxicity toward extraovarian tissue, induces ovarian aging analogous to natural ovarian aging.^32^ Researchers determined that there were significant differences in both the alpha and beta diversity between the mice in the VCD group and the control group.^33^ The levels of several genera, including *Helicobacter*, *Odoribacter*, and *Alistipes*, were lower, and Clostridium XIVa, *Barnesiella*, *Bacteroides*, and *Mucispirillum* were higher among the dominant microbiota of the VCD group.^33^ Studies in *Drosophila* revealed that the intestinal epithelium deteriorates as females age, which leads to high microbiota loads, barrier impairment, and decreased lifespan.^34,35^ The steroid hormone ecdysone, produced by active ovaries of the fly, could stimulate the division and expansion of intestinal stem cells and enhance their reproductive output.^36^

To further probe the more complicated and deeper causal linkage between the gut microbiota and natural ovarian aging, fecal microbiota transplantation (FMT) was applied to naturally aging mice. Aged mice (42 weeks old) treated with FMT from young donors (5 weeks old) presented a “young animal-like phenotype” in the gut microbiome, and follicular atresia and apoptosis were reduced to improve the fertility of aged mice.^37^ These ovarian aging-delay effects might be explained by improving the inflammatory status and immune microenvironment.^37,38^ Substantial evidence in aged zebrafish also demonstrated the effect of the young microbiota on reproductive modulation. FMT from young donors to aged zebrafish could counteract the adverse effects of subsequent exposure to toxic pollutants, potently reduce the levels of membrane-bound progestin receptor transcripts, promote the progression of oogenesis, and reduce the rate of malformations caused by toxic pollutants, and their offspring presented a stronger locomotor capability and swam much faster.^39^ Overall, maintenance of a “youthful” gut microbiota during ovarian aging may limit ovarian senescence and provide additional benefits.

## The gut microbiota and premature ovarian insufficiency

Rather than natural ovarian aging, some women in the population experience a premature decline in ovarian function, that is, premature ovarian insufficiency (POI).^40^ POI is a clinical syndrome defined by exhaustion of normal ovarian activity in patients younger than 40 years, which presents as a menstrual disorder (amenorrhea or oligomenorrhea) with elevated gonadotropin levels and low E2 levels.^41^ The estimated global prevalence is as high as 3.7%, while 0.1% of women aged less than 30 and 0.01% of women aged less than 20 present with the condition.^42^ POI has attracted public attention because of its adverse effects on fertility and subsequent negative health impacts, such as increased risk of cardiovascular diseases, cognitive impairment, mood and sexual dysfunction, osteoporosis and shorter life expectancy.^43–45^ POI is a highly heterogeneous condition, with 70 to 90% of POI cases having unknown causes in addition to known genetic, autoimmune, infectious, and iatrogenic factors, that is idiopathic POI.^42^ Recently, mounting evidence has suggested that gut microbiota dysbiosis is associated with POI. In this section, we review the current research linking gut microbiota and POI, focusing on idiopathic POI, iatrogenic POI and POI-driven diseases.

## Idiopathic POI

It has been noted that there was a reduction in beta diversity in patients with POI.^46^ At the phylum level, higher *Bacteroides* and lower *Firmicutes* abundances were observed in POI patients, while higher *Butyricimonas*, *Dorea*, *Lachnobacterium*, and *Sutterella* and lower *Bulleidia* and *Faecalibacterium* abundances at the genus level were observed in women with POI (*n* = 35) than in healthy women (*n* = 18).^47^ These microbial changes were correlated with the levels of FSH, LH, E2, and AMH and the FSH/LH ratio.^47^ In addition, decreases in the abundances of the genera *Eggerthella* and *Staphylococcus* and increases in the abundances of the genera *Comamonas* and *Barnesiella* were observed in POI patients (*n* = 10) relative to controls (*n* = 10).^46^ Researchers have also revealed positive associations between *Eggerthella* and several serum metabolites.^46^ Although POI shares similar clinical manifestations with natural ovarian aging, few studies have reported differences in the gut microbiota between them.

Hormone replacement therapy (HRT) is the primary recommended treatment given to women with POI both for symptom management and disease prevention.^40^ Jiang et al. determined that the alterations in beta diversity, relative abundance of the genus *Eggerthella*, serum metabolites, and E2 and progesterone hormone levels related to POI were significantly reversed after HRT implementation,^46^ which further validated the interaction between estrogen and gut microbiota. Animal experiments confirmed that *E. lenta* gavage induced ovarian fibrosis in mice and that estrogen treatment alleviated ovarian fibrosis.^46^ Despite the potential linkage between POI and the gut microbiota, the causal nature of this relationship still needs to be deeply investigated.

Taken together, these results show that gut microbiota dysbiosis, such as disturbance of core microbiota (*Lachnoclostridium* and *Bacteroides*), short-chain fatty acid (SCFA) producers (*Bacteroides* and *Faecalibacterium*) and an increase in opportunistic pathogenic bacteria (*Eggerthella*), may be involved in the pathology of POI, and the reversal of gut microbiota may be a new strategy for ovary preservation to some extent.

## Iatrogenic POI

As noted above, most POI cases are idiopathic. However, among the few cases with known causes, iatrogenic factors account for a large proportion.^48–50^ Many medical factors can damage ovarian function, such as pelvic radiotherapy, chemotherapy and surgery, in tumor patients, which can lead to premature decline or even total loss of ovarian function.^51^ Specifically, the higher the therapeutic dose, the larger the radiation area and the larger the surgical area, the greater the impact on ovarian function.^52–55^ Patients who have undergone ovarian surgery, especially laparoscopic surgery, are candidates for iatrogenic POI.^48^ Bone loss and genitourinary atrophy are two major challenges faced by many young cancer patients and postsurgical women due to iatrogenic ovarian injury, which seriously impacts their overall quality of life.^50^

A study profiled and analyzed the changes in gut microbiota in ovarian cancer patients who underwent different anticancer treatments and found that postsurgery samples exhibited a significant decrease in the abundances of the phyla *Bacteroidetes* and *Firmicutes* and a significant increase in the abundance of the phylum *Proteobacteria* compared with presurgery samples; however, after-chemotherapy samples exhibited significantly increased abundances of the phyla *Bacteroidetes* and *Firmicutes* and a decreased abundance of the phylum *Proteobacteria* compared with before.^56^

Bilateral ovariectomy is the most mature method to build an iatrogenic menopause animal model and has been widely used in menopause and menopause-related research, such as menopausal osteoporosis and genitourinary atrophy.^53–59^ Therefore, we focused on mouse ovariectomy studies to demonstrate the relationship and mechanism of the gut microbiome and iatrogenic menopause. Animal studies have shown that changes in the gut microbiota are associated with iatrogenic ovarian aging and associated relevant adverse outcomes.

**Figure 2.**
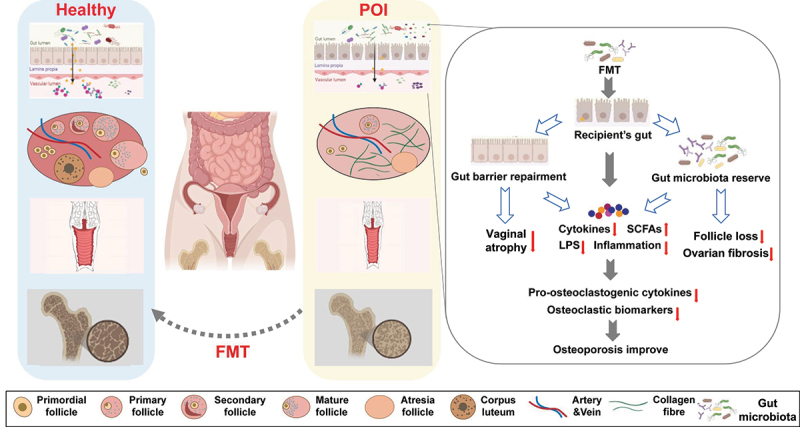
FMT alter the gut microbiota and slow the progression of ovarian aging related diseases.

Many studies based on an ovariectomized (OVX) mouse model have revealed that the gut microbiota could be affected by iatrogenic menopause.^60^ Significant differences in beta diversity^61–64^ and alpha diversity^62^ have been observed between the gut microbiota of the OVX group and the sham group. Several studies found that the OVX group had a higher *Firmicutes*/*Bacteroidetes* ratio and lower relative abundance of *Deferribacteres* at the phylum level.^65–68^ At the genus level, the abundance of the predominant genera in the sham group decreased significantly in the OVX group (87% vs. 29%).^62^ In contrast, lower-abundance genera in the sham group were importantly enriched in the OVX group (<5% vs. 63%).^62^ Zhang et al. identified four key significant genera in OVX rats: *Incertae_Sedis, Anaerovorax, Anaerotruncus* and *Helicobacter*.^63^ Furthermore, the OVX group exhibited apparent weight gain,^65^ intestinal barrier impairment^61^, bone loss^65,66^ and vaginal atrophy.^64^ The gut microbiota is indispensable for OVX-induced osteoclast differentiation, and germ-free (GF) animals were protected against osteoporosis induced by OVX. The *Firmicutes Bacteroidetes* phylum and the *Firmicutes/Bacteroidetes* ratio were critical in osteoclast differentiation, and supplementation with the probiotic *Lactobacillus salivarius LI01* from the *Firmicutes* phylum prevented OVX-induced osteoporosis in mice.^67^ FMT from ovary-intact mice inhibited the gut microbiota changes and weight gain induced by OVX, suppressed the release of pro-osteoclastogenic cytokines, increased fecal SCFA levels (mainly acetic acid and propionic acid), reduced intestinal permeability and ultimately prevented OVX-induced bone loss.^65^ Surprisingly, Huang et al. showed that FMT from ovary-intact fecund females changed the gut microbiota and significantly alleviated vaginal epithelial atrophy in OVX mice.^64^ In general, FMT might act as a promising target for the prevention and treatment of iatrogenic ovarian aging-related consequences (Figure 2).

## Association among antiaging interventions, ovarian function and intestinal microbes

### Antiaging drugs

To date, no clinically feasible techniques are available to either delay or reverse ovarian dysfunction associated with advanced age.^69^ However, important advances have been made in the field of anti-ovarian aging during the past decades, and numerous emerging antiaging agents, such as coenzyme Q10 (CoQ10), melatonin, nicotinamide mononucleotide (NMN), resveratrol, rapamycin and N-acetyl-L-cysteine (NAC), have shown great potential to delay or reverse ovarian aging.^68–70^ Although no study has directly explored the relationship among these antiaging drugs, ovarian function and gut microbiota, some indirect evidence has shown that the beneficial effects of these drugs may be associated with gut microbes. As shown in Table 1, these drugs may work through intestinal microbes.^102^

**Table 1.** Association among antiaging drugs, ovarian function and intestinal microbes.

Drug	Animal	Gut microbiota alterations induced by antiaging drugs	Effect on ovarian function
Resveratrol	Hen^71^ Mouse^72–75^ Rat^76^ Annual fish N. guentheri^77^	*Firmicutes, Rikenellaceae, Lactobacillus, Lactobacillales, Firmicutes/Bacteroidetes* ratio, *Blautia, Bacteroides, Lachnospiraceae_NK4A136_group, Lachnoclostridium, Parabacteroides, Ruminiclostridium_9*↑ *Clostridiales_bacterium_CHKC1001, Lactobacillus_mucosae, Desulfovibrio, Lachnospiraceae_NK4A136_group*↓	Egg-laying rate reduction, atretic follicles↓ E2, FSH, AMH, ovarian relative weight, primary and growing follicles, oocyte quantity and quality, litter size, oocyte primary growth, cortical alveolus and vitellogenesis stage↑
Melatonin	Mouse^78–80^ Hen^81^	Alpha diversity, *Bacteroides, Alistipes, Firmicutes, Firmicutes/Bacteroidetes ratio*↓ *Lactobacillales, Lactobacillus, Bacteroidetes*↑	E2, LH, growing follicles, pool of follicles, litter size, oocyte quantity and quality↑
NMN	Mouse^82–84^	*Firmicutes, Firmicutes/Bacteroidetes ratio*↓ *Bacteroidetes*↑	Oocyte quality, number of follicles at different stages, corpora luteum, estrus cycle↑ FSH↓
Rapamycin	Mouse^85–88^ *Drosophila* ^89^	*Segmented filamentous bacteria (Candidatus arthromitus sp.) ↑* *Bacterial load, Alphaproteobacteria↓*	Ovarian reserve, oocyte quality↑ Disturbed estrous cycle but recovered after 2 months
CoQ10	Rat^90^ Mouse^91–93^ Human^94,95^ Hen^96^ Sow^97^ Red tilapia^98^	*Ruminococcus, Lachnospiraceae AC 2044, Lactobacillus, Bifidobacterium↑* *Helicobacter, Proteobacteria↓*	Primordial, preantral, antral follicles, ovarian response to stimulation, numbers of zygotes retrieved, embryological parameters, pregnancy↑
NAC	Mouse^99–101^	*Akkermansia, Bifidobacterium, Lactobacillus, Allobaculum↑* *Desulfovibrio, Blautia↓*	Oocyte quality, total blastocyst cell number, litter size↑

(↑, increase; ↓, decrease).

E2, estradiol; FSH, follicle-stimulating hormone; LH, luteinizing hormone; AMH, anti-Müllerian hormone; CoQ10, coenzyme Q10; NAC, N-acetyl-L-cysteine.

#### CoQ10

CoQ10 is a fat-soluble, vitamin-like benzoquinone compound, plays a critical role in cellular energy production and functions as an antioxidant in its reduced form.^69^ The synthesis of CoQ10 appears to decrease with age, which coincided with the time point of fertility and oocyte quality decline.^103^ Pretreatment with CoQ10 during the fertility treatment improved ovarian response and the quality of embryo in young patient with diminished ovarian reserve.^104^ CoQ10 treatment in mice not only preserved the ovarian follicle pool but also promoted ovulation of gametes capable of supporting normal development.^68,103^ Dietary CoQ10 supplementation also had beneficial effects on the reproductive variables of aged hens^105^ gestating sows with high parity^91^ and red tilapia.^82^ However, in women with infertility undergoing assisted reproductive technology procedures, CoQ10 increased clinical pregnancy but showed no effect on live birth or miscarriage rates.^106^ CoQ10 also has an impact on the gut microbiota and the subsequent biomarkers it produces. Three-week of administration of CoQ10 resulted in 2.4 times and 7.5 times increase in the relative abundance of *Ruminococcus* and *Lachnospiraceae AC 2044 groups*, respectively, as well as a 1.26-fold increase in butyrate.^77^ Pumpkin juice rich in CoQ10 gavage increased the abundance of *Lactobacillus* and *Bifidobacterium* and further protected the gut barrier by reducing *Proteobacteria* of mice.^72^

#### Melatonin

Melatonin is an indoleamine produced by all cells, especially those in the pineal gland.^76^ As a powerful antioxidant, melatonin has been shown to delay the decline in fertility in female animals.^71,73^ Oxidative stress (OS) is considered a key factor in ovarian aging that accelerates follicle loss and atresia,^74^ along with significant gut microbiota dysbiosis^73,107^ while melatonin reverses OS-induced phenotypes and improves gut microbiota communities.^75^ Melatonin treatments in mice not only significantly increased the number and quality of oocytes but also led to more blastocyst generation after in vitro fertilization and larger litter sizes than those in the controls.^73^ Hao et al. identified that melatonin might increase the levels of E2 and LH as well as the levels of immunoglobulin and the numbers of growing follicles in aged laying hens.^71^ Melatonin pretreatment reduced the Firmicutes abundance and the *Firmicutes/Bacteroidetes* ratio and increased the content of *Bacteroidetes* at the phylum level.^108^ Additionally, oral melatonin supplementation significantly reversed gut microbiota dysbiosis induced by a high-fat diet (HFD), including the decreased alpha diversity of the intestinal microbiota, the decreased relative abundances of the genera *Bacteroides* and *Alistipes*, and the increased relative abundances of the order *Lactobacillales* and the genus *Lactobacillus*.^109^ The marked correlation between acetic acid production and the relative abundances of the genera *Bacteroides* and *Alistipes* after FMT from melatonin-treated mice indicated that gut microbiota reprogramming might be the potential mechanism of melatonin treatment.^109^

#### Nmn

NMN is another promising anti-ovarian aging drug. The loss of oocyte quality with reproductive aging accompanies declining levels of nicotinamide adenine dinucleotide (NAD+),^81^ and as an intermediate of NAD+ biosynthesis, NAD+ can be compensated by NMN supplementation to play a role in delaying ovarian aging.^107^ Twenty-week oral administration of NMN to middle-aged mice improved the estrous cycle condition, increased the number of follicles at different stages and the corpora lutea and serum FSH, and enhanced mitochondrial biogenesis, autophagy, and protease activity of granulosa cells (GCs) compared with the control treatment.^110^ In HFD-induced POI mice, NMN administration reduced ovarian inflammation and the adipose size of abdominal fat tissue and improved oocyte quality.^70^ NMN treatment increased the abundance of butyrate-producing bacterial genera, such as *Ruminococcae_UCG-014, Prevotellaceae_NK3B31_group* and the probiotic *Akkermansia muciniphila*, while decreasing the abundances of several harmful bacterial genera, such as *Oscillibacter* and *Bilophila*.^78^ NMN also reduced intestinal mucosal impairment and maintained mucosal barrier integrity.^78^

#### Resveratrol

Resveratrol, a natural polyphenol, potentially delays ovarian aging and protects against age-associated infertility^79,111,112^ and is capable of restoring the microbiota composition and reversing intestinal dysbiosis.^83^ A study revealed that resveratrol ameliorated the oxidative stress-induced reduction in egg-laying rate and serum hormone level changes, as well as gut microbiota dysbiosis, and elevated the concentrations of the main SCFAs in laying hens.^84^ This study directly proved that resveratrol modulated ovarian function through intestinal microbes. Enrichment of *Bacteroides, Lachnospiraceae_NK4A136_group, Blautia, Lachnoclostridium, Parabacteroides* and *Ruminiclostridium_9*, collectively referred to as the resveratrol-microbiota, displayed anti-obesity functions.^113^

#### Rapamycin

The mammalian target of rapamycin (mTOR) signaling pathway exerts a vital role in folliculogenesis in female germline cells.^114^ Activation of the AKT/mTOR signaling pathway in POI patients has been reported.^115^ Suppressing mTOR signaling in HFD-induced POI mice reversed follicle loss and extended the ovarian lifespan.^116^ Rapamycin is a natural macrolide compound initially segregated from bacteria and serves as a TOR signaling inhibitor.^85^ Rapamycin treatment increased ovarian reserve^86^ and prolonged ovarian lifespan with oocyte quality improvement, especially in aged mice.^87^ In a cyclophosphamide-induced POI model, rapamycin prevented primordial follicle loss and protected the ovarian reserve.^88^ A significant increase in the levels of segmented filamentous bacteria (Candidatus Arthromitus sp.) in rapamycin-treated mice was observed.^89^ However, although rapamycin rescued intestinal barrier dysfunction and increased microbial loads in Drosophila, its antiaging effects seemed to be independent of the gut microbiota.^92^

#### Nac

NAC is synthesized to help stimulate the synthesis of glutathione to exert antioxidant effects.^94^ A study conducted in mice proposed that the administration of NAC could improve the quality of preimplantation oocytes and increased the total blastocyst cell number and litter sizes, thus delaying the fertility decline with reproductive aging.^96^ NAC alleviated gut dysbiosis in HFD-fed mice by promoting the growth of several beneficial bacteria and affect the metabolic pathways of intestinal bacteria.^97,98^ In general, evidence on the effects of NAC treatment on reproductive aging and gut microbiota are rare and fertility-related researches to assess the effects of NAC administration are needed.

## Antiaging diet

In addition to antiaging drugs, antiaging diets seem to be a more popular option, of which caloric restriction (CR) is currently regarded as the most reproducible strategy.^90,95^ CR refers to reducing energy intake without incurring malnutrition or a lack of essential nutrients.^93,117^ As a dominant paradigm for antiaging diets, its life-extending effect has been widely acknowledged.^99^ Over the past decades, attention has been given to the effect of CR on reproduction and gut microbiota (Tables 2 and 3).

**Table 2.** Caloric restriction modulates ovarian function.

Dietary regimen	Animal	Effect on ovarian function
10-week 45% CR	Rat^118,119^	Ovarian reserve, total follicles, surviving follicles↑ LH, FSH, E2, atretic follicles ↓ Irregular estrous cycles↑
26-week 30% CR	Mouse^120^	Ovarian reserve, surviving follicles↑ Atretic follicles↓ Irregular estrous cycles↑
9-month CR (Alternate-day feeding)	Mouse^121^	ER/AR↑
93-day 30% CR	Mouse^85^	Ovarian reserve↑
2-month 25% and 45% CR	Rat^122^	Ovarian reserve, healthy follicles↑
24-week 10% CR	Mouse^123^	Ovarian reserve, pregnancy rate↑ Respond to superovulation↓

(↑, increase; ↓, decrease).

CR, caloric restriction; E2, estradiol; FSH, follicle-stimulating hormone; LH, luteinizing hormone; AMH, anti-Müllerian hormone; ER, estrogen receptor; AR, androgen receptor.

**Table 3.** Gut microbiota alterations induced by caloric restriction.

Dietary regimen	Animal	Gut microbiota alterations
4-week VLCD	Human^124^	*Anaerostipes hadrus, Blautia sp., Ruminococcus faecis, Bifidobacterium sp*.↑ *Agathobacter rectalis*↓
30-day 40% CR	Mouse^125^	*Lactobacillaceae, Erysipelotichaceae, Bacteroidaceae, Verrucomicrobiaceae*↑ *Firmicutes*↓
28-month 30% CR	Mouse^126^	*Firmicutes, Peptostreptococcaceae, Clostridium sensu stricto 1, Turicibacter*↓ *Actinobacteria, Verrucomicrobia, Bifidobacterium, Lactobacillus*↑
2-week 30% CR	Mouse^127,128^	*Lactobacillus*↑
3-month 34% CR	Human^129^	*Subdoligranulum, Collinsella*, ↓ *Parabacteroides, Alistipes, Bacteroides*↑
8-week VLCD	Human^130^	Bacteria capable of foraging on host glycans (*Akkermansia*) ↑ Bacteria specialized for the metabolism of plant polysaccharides (*Roseburia, Ruminococcus, Eubacterium*) ↓
4-day, 70% protein-restricted, 30% CR	Human^131^	Butyrate producers (*Faecalibacterium prausnitzii, Roseburia intestinalis*) ↓
8-week 30% CR	Rat^132^	*Lactobacillus*↑
62-week 30% CR	Mouse^133^	*Lactobacillus*↑
Lifelong 30% CR	Mouse^134^	*Lactococcus*↓ *Lactobacillus, Bifidobacterium*↑
8-week 30% CR	Mouse^135^	*Helicobacter pylori*↓ *Lactobacillus, Bifidobacterium*↑
2-month 30% CR	Mouse^136^	*Clostridia, Clostridiales, Firmicutes*↓
45-day 25% CR	Mouse^137^	*Firmicutes/Bacteroidetes*↓ *Bacteroidetes*↑
1-year 30% CR	Human^138^	*Firmicutes/Bacteroidetes↓* *Bacteroides, Roseburia, Faecalibacterium*, *Clostridium↑*
12-week VLCD	Human^139^	*Bacteroides↓* *Firmicutes, butyrate-producing bacteria↑*

(↑, increase; ↓, decrease) CR, caloric restriction; VLCD, very-low-calorie diet.

Obesity, excess body fat and insulin resistance have detrimental effects on ovarian follicle development, thereby damaging the reproductive capacity of female animals.^100,101,140–143^ CR was associated with weight loss, reduced abdominal visceral fat accumulation, and increased insulin sensitivity.^86,144^ Consequently, imposed ovarian aging-delay effects through the inhibition of follicle loss and preservation of the follicle pool. Unlike in control rodents, rodents that underwent CR treatment showed significantly increased numbers of primordial follicles and inhibited development of follicles at different stages^86,118,120,122,123,145^ as well as an increased ratio of estrogen to androgen receptors.^119^ Ten weeks of CR feeding decreased serum LH, FSH, and estrogen levels.^120^ CR exerted a similar effect in HFD-induced POI mice.^116^ The p53 downregulation induced by CR may inhibit follicle atresia because of its effects on the cell cycle and apoptosis.^145^ CR can also rescue age-related microbiota alterations observed in aged female mice.^121^ Three months of 34% CR helped to attain significant weight loss, and decreased waist circumference coincided with changes in five bacterial genera (*Subdoligranulum, Collinsella, Parabacteroides, Alistipes*, and *Bacteroides*).^146^ Thirty days of 40% CR in mice increased the abundances of *Lactobacillaceae, Erysipelotrichaceae, Bacteroidaceae* and *Verrucomicrobiaceae* and decreased the abundance of *Firmicutes* at the family level, improved glucose metabolism, fat browning, and cold tolerance and reduced LPS-binding protein and circulating LPS levels.^129^ In addition, severe CR decreased body fat deposition and improved glucose tolerance^125^ but enriched *Akkermansia* which capable of foraging on host glycans and expensed *Roseburia, Ruminococcus*, and *Eubacterium* which specialized for the metabolism of plant polysaccharides in human feces.^147^

Chronic inflammation is another common condition in aging^130^ as well as in ovarian aging.^38^ Inflammation might be one of the mechanisms contributing to reproductive aging. Lliberos et al. reported that the increase in the intraovarian percentage of B cells, CD4^+^ T cells and macrophages was consistent with the process of ovarian aging and that age-dependent overexpression of multiple proinflammatory cytokines, including IL-6, TNF-α, and IL-1α/β, and the inflammasome genes NLRP3 and ASC was observed during reproductive aging.^148^ Inhibition of the NLRP3 inflammasome helps to prevent ovarian aging.^38^ The gut microbiota plays a vital role in the modulation of the inflammatory response.^149,150^ CR might delay immune senescence by shaping the gut microbiome. CR-microbiota transplantation increased alpha diversity and lowered the abundances of *Clostridium ramosum, Hungatella hathewayi*, and *Alistipes obesi* in recipients, and these CR-related microbiota reduced the levels of intestinal and hepatic effector memory CD8^+^ T cells, intestinal memory B cells, and hepatic effector memory CD4^+^ T cells.^125^ Another study discovered five other CR-related operational taxonomic units in obese women: *Anaerostipes hadrus*, *Blautia sp., Agathobacter rectalis, Ruminococcus faecis* within *Ruminococcaceae*, and *Bifidobacterium sp*. within the family *Bifidobacteriaceae*, and reduced systemic inflammation was found in obese women after CR.^151^ Moreover, CR for 2 weeks dramatically reduced intestinal inflammation and increased the protective intestinal microbiota taxon *Lactobacillus* and the survival rate of 2-month-old female mice after lethal-dose methotrexate exposure.^124^ The increased *Bifidobacterium* and *Lactobacillus* levels induced by lifelong CR were inversely correlated with the levels of bile acids, including ChDxCA and secondary bile acids (DxCA/TDxCA and a ChDxCA derivative).^128^ The *Lactobacillus*-dominant microbial community promoted by CR decreased the levels of systemic microbial antigens and inflammatory markers.^126^ Based on the discovery of several molecular pathways associated with CR mentioned above, CR might be a possible strategy to improve inflammation and possibly promote obesity-related infertility and reproductive span.

Notably, these anti-aging interventions described above either examined the effects on the gut microbiome or ovarian function, and few studies except one^84^ speculated on anti-aging drugs, the gut microbiome, and ovarian function simultaneously. Thus, the current evidence that the gut microbiome involved in the mechanism of treatment affects ovarian function is fragmented and scattered, which is also a limitation of this review. There is still a large gap in how these antiaging interventions interact between the gut microbiota and ovarian function. Thus, more research focusing on the effects of these interventions on the gut microbiome and ovarian function is urgently needed to link the interaction of the three parts.

## Conclusions

In conclusion, altered composition and function of the gut microbiota play an important role in the pathogenesis of reproductive aging. Experimental and clinical studies have uncovered the relationship between gut dysbiosis and follicle development, as well as a disturbed immune response. Results from FMT studies provide a new insight to anti-ovarian aging, that is the maintenance of youthful gut microbiota helps to preserve ovarian function and prevent ovarian-related diseases. Microbiota-based intervention to delay or reserve ovarian aging is an appealing approach and may offer new therapeutic strategies for intestinal microbiota regulation to improve female fertility.

Furthermore, investigation of antiaging interventions such as antiaging drugs and CR may improve the gut microbial imbalance and promote a healthier intestinal ecological environment. However, evidence from the current scientific literature cannot offer direct conclusions regarding these measures. The majority of the relevant studies were conducted in animal models, which cannot simply apply to human beings. Therefore, future studies should shift from simple correlation analysis to large-scale cohort research and focus on the potential causes and underlying mechanisms to verify the beneficial effects of these interventions in ovarian aging.

Given the wide alterations in the gut microbiota composition and function throughout ovarian aging, it has been suggested that the gut microbiota may be suitable for deciphering the processes of expected and unexpected ovarian aging in women. Imbalance in the gut microbiota may lead to the progression of various ovarian aging-related conditions. Although ovarian aging is unavoidable, maintenance of a balanced gut microbiota is a potential way to delay ovarian aging and subsequent adverse outcomes.

## Perspectives

Recent studies have achieved considerable progress in elucidating the roles of the gut microbiota in ovarian aging. However, it is still unclear whether gut microbial changes are the cause or consequence of ovarian aging, and the exact time point at which to modulate the gut microbiota to exert anti-ovarian aging effects remains unsolved. Understanding the relationship between the gut microbiota and ovarian aging is still relatively preliminary, the core mechanism needs to be further explored, and more high-quality evidence is urgently needed. Many factors may affect the gut microbiota, such as age, obesity, dietary pattern, antibiotic usage, and smoking, and these confounders need to be fully considered in future studies. Additionally, metagenomic analyses with a strain-level resolution are warranted because of the high variability of specific species and strains.

Based on the current scientific literature, we can only find some bacterial groups that are closely related to ovarian aging, and the current evidence is relatively scattered. The effects are likely achieved through a pleiotropic mechanism, one of which is an altered production of microbe-associated metabolites.^127^ The role and molecular mechanism of specific strains and their metabolites in follicle development are still uncertain and remain largely unexplored. Several studies have reported that both intestinal and serum metabolite disorders exist in POI samples,^46,152,153^ and some metabolites may mediate the function of the hypothalamic-pituitary-ovary axis.^152,154^ Significant correlations among SCFAs with follicular development and follicular fluid hormones were found.^16^ Supplementation with specific metabolites may be another choice for regulating ovarian function. Zhang, et al found that polyamine metabolite spermidine level was reduced in ovaries of aged mice and supplementation with spermidine promoted follicle development, rejuvenated oocyte quality and improved fertility rate of aged mice.^155^ And Guo, et al found that Branch chain amino acid (BCAA) insufficiency could lead to POI, and supplementation with BCAA ameliorated ovarian dysfunction from reactive oxygen species-induced POI in mice.^152^ Long-term NMN treatment led to significantly higher levels of bile acid-related metabolites.^78^ However, there are still a series of specific metabolites should be further identified and validated. In addition, follicular atresia is initiated with the apoptosis of GCs after birth.^156^ A study has indicated that the gut microbiota may participate in regulating ovarian follicular development via SCFAs affecting GC apoptosis.^16^ The effects of other certain metabolites to GC apoptosis are worthy exploring.

Furthermore, numerous studies have suggested that bacteriotherapy using three slightly different agents: probiotics, prebiotics, and synbiotics are promising for the prevention and treatment of human general aging and age-related disorders,^5,103,157–161^ in which *Lactobacillus* and *bifidobacterium* are the most commonly used, though the effects varied across studies, which depending on dosage, duration, and their components.^162^ Several clinical trials have demonstrated that the intake of a probiotic mixture decreasing inflammatory levels, increased the abundance of an anti-inflammatory commensal bacterium and improved cognitive function in older people.^80,131^ Additionally, studies have shown that some probiotic strains have beneficial effects on male reproductive dysfunction.^132,133^ Supplementation of *Lactobacillus* and *Bifidobacterium* improved sperm motility and reduced rate of DNA fragmentation in males.^134^ Supplementation with probiotics can restore serum testosterone levels and increased spermatogenesis in aging mice.^135^ However, the effects of the probiotics, prebiotics, and synbiotics on the ovarian aging are unclear, and more studies are required to prove whether they could be effective strategies to counteract ovarian aging.
